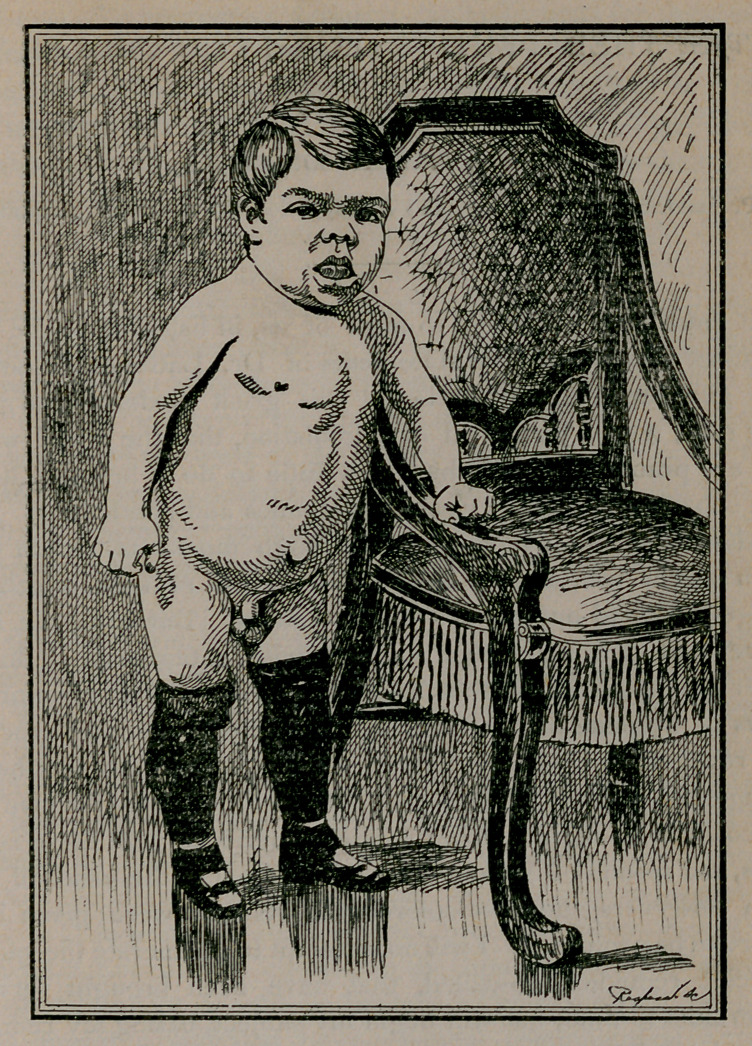# A Case of Cretinism

**Published:** 1893-02

**Authors:** Hugh Hagan

**Affiliations:** Atlanta, Ga.


ATLANTA
MEDICAL AND SURGICAL JOURNAL.

VOL. IX. FEBRUARY, 1893. NO. 12.

EDITED BY
LUTHER B. GRANDY, M. D., AND MILLER B. HUTCHINS, M. D.,
WITH THE CO-OPERATION OF
H. V. M. MILLER, M. D., LL. D., VIRGIL O. HARDON, M. D., AND
FLOYD W. McRAE, M. D.

### ORIGINAL COMMUNICATIONS.

### A CASE OF CRETINISM.

BY HUGH HAGAN, M. D.,

ATLANTA, GA.

C., aged twelve years, was brought to my office to be treated for nervousness.

The mother of the boy gives a good family history and is herself remarkably healthy. She has four other children, all of whom are healthy and sound minded. The father is a sober, healthy man with good family history, no mental, nervous or hereditary diseases known. Both parents of subject are of French extraction. This child was born in Georgia and reared in Atlanta. The child has been shown to many medical men, both regular and irregular practitioners. The only diagnosis given by any of them was, "the boy was a freak." The boy's condition was so typical of cretinism that I did not hesitate to diagnosticate that condition, and so stated to the parents. The boy is thirty-one inches tall, twenty-one inches around the head; from root of nose to occipital protuberance, thirteen inches; from ear to ear over the crown of head, eleven inches; twenty-three inches around the chest; arm and fore-arm, six inches in circumference; thighs, eleven inches; and legs, nine inches; weight, forty pounds. His countenance looks like an old man, heavy, dull and expressionless; the nose flat, broad and fleshy; mouth open, lips thick, and the tongue twice as large as normal, filling the buccal cavity, making it impossible to swallow solid food, necessitating a liquid or semi-solid diet. The protrusion of tongue possible only so far as lips. The neck is short and beefy; skin dull, dry and very thick; chest remarkably large, and the general appearance of limbs like those of a well-developed dwarf athlete. There is a marked lordosis, with consequent protrusion of abdomen. A large umbilical hernia is also present. The genitals are large and well developed.

His special senses are intact. His intelligence not more that of a child of two or three years. His vocabulary is very limited. He is apathetic, and non-excitable. Has never had any marked frenzy. He understands the ordinary questions put to him, but never acts in the initiative. He cannot speak in sentences, and has to be looked after as an infant. His ability to walk is feeble, ten or fifteen steps by himself is about the longest distance he can make unassisted without falling, though if given the hand of the nurse walks well. He is unable to stand alone with feet together, but does not fall in any particular direction. The fundi oculi are healthy. The vegetative organs sound.

The only symptom or deformity of typical alpine cretinism that is wanting in this case is goitre. The thyroid does not seem to be affected.

I cite this case because no telluric, or paludal, or hereditary etiology can be found, the child being born of healthy parents, in a healthy country, and that country non-mountainous. The only other case of cretinism I ever saw in North America was a quaker child in Philadelphia. The rarity of these cases of cretinism will be appreciated when such an experienced man as Spitzka only recounts three cases, and those not true cases of cretinism. The accompanying photograph will give a good idea of the little fellow.

## Figures and Tables

**Figure f1:**